# Experimental assessment of dry water materials for inhibiting explosion under the coal gasification atmosphere

**DOI:** 10.1098/rsos.241395

**Published:** 2025-02-12

**Authors:** Guangqian Liang, Peikai Luo

**Affiliations:** ^1^ Intelligent Architecture Institute, Zhejiang College of Security Technology, Ouhai Avenue 2555, Wenzhou 325000, People's Republic of China; ^2^ Zhejiang Provincial Innovation Center of Laser Intelligent Equipment Technology, Wenzhou, 325000, People's Republic of China

**Keywords:** coal dust, explosion characteristics, hybrid mixture, dry water material, inhibitory mechanism

## Abstract

This study focused on the inhibitory effects of dry water materials on the coal dust explosion characteristics in the coal hydrogenation process by adopting a 20 l explosion device. Additionally, the inhibitory effects of dry water material were compared with SiO_2_ and NH_4_H_2_PO_4_. The results showed that 10 wt% dry water materials notably delay the arrival time of the peak explosion pressure, significantly inhibiting the initial energy release during the explosion, resulting in the maximum rate of explosion pressure rise decreasing by 61.96%. As the dry water material adds up to 50 wt%, the maximum explosion pressure significantly reduced to 0.258 MPa and delayed by 431.1 ms. The flame propagation velocity reduced from 2.19 to 0.33 m s^-1^. In addition, dry water material, with its excellent heat absorption and adsorption and isolation properties, had more significant inhibitory effects than SiO_2_ and NH_4_H_2_PO_4_.

## 1. Introduction

In the global energy structure, coal still occupies a prominent position. However, amidst the intensifying energy crises and mounting challenges of environmental pollution, coal hydrogen gasification technology emerges as a groundbreaking solution for converting fine-grade coal. This technology efficiently and cleanly converts coal resources into natural gas while simultaneously yielding high-value light aromatic hydrocarbons as by-products. It holds great importance in driving the adjustment of energy structures and safeguarding environmental protection [1,2]. Nevertheless, under the condition of medium temperatures and high pressures, the rapid reaction between coal powder and hydrogen generates a gas-powder accompanied by methane, light hydrocarbons and clean semi-coke. The potential explosion risk cannot be ignored and has become a key issue in industrial applications. To effectively contain this risk and ensure production safety, a systematic study of dust explosion mechanisms under various conditions is essential for developing targeted preventive measures and control methods. These efforts will reduce the risk of gas-powder mixture explosions at their source while providing solid support for the safe, stable and efficient operation of coal hydrogen gasification technology.

The high-pressure and temperature environment accompanies strong shock waves and rapidly expanding flames generated by the explosion, triggering a chain reaction in areas with dust accumulation and leading to sustained explosive phenomena. Cashdollar’s research [3] clearly identified vital parameters for coal dust explosion characteristics: maximum explosion pressure (*P*_max_), maximum rate of pressure rise ((d*P*/d*t*)_max_) and flame propagation velocity (*v*). Furthermore, Li *et al*. [4] focused on the impact of concentration on coal dust explosion performance, revealing that nearly all coal dust exhibits optimal explosive efficiency at a concentration of approximately 250 g m^−^³. This finding provides crucial data support for optimizing coal dust handling processes. The research conducted using a 20 l spherical vessel on explosion characteristics of CH_4_/H_2_/coal dust/air mixtures revealed that the addition of methane significantly enhances the *P*_max_ of low-volatile coal dust. The increase in hydrogen generation further promotes the elevation of maximum explosion pressure, particularly during self-heating [5]. Additionally, significant progress has been made in studying the influence of H_2_ content on explosive characteristics [6,7]. The research results indicated that as the proportion of hydrogen increases, both explosion pressure and flame propagation velocity exhibit an upward trend accompanied by an increase in flame temperature, which is of great significance for evaluating the safety and optimizing application conditions for hydrogen-containing fuel systems.

Given the unique nature of dust explosions, current research focuses on extinguishing materials such as gases [8–10], porous materials [11,12], fine water mist, powders and multiphase composite materials [13]. Among them, the inertization technology as a core strategy injects inhibitors into combustible dust to increase its minimum ignition energy and enhance safety. Jiang *et al*. [14] demonstrated that the addition of NaHCO_3_ and NH_4_H_2_PO_4_ powder inhibitors effectively consumes the key free radicals in the flame, resulting in a significant reduction in both flame velocity and temperature during dust explosions. Janes *et al*. [15] pointed out that common powder inhibitors, such as inert rocks and expandable graphite, exhibit strong suppression capabilities through physical effects like thermal cooling, dilution, radiation absorption, turbulence modification and limitation of oxygen diffusion. Furthermore, Chen *et al*. [16] experimentally demonstrated the distinct role of SiO_2_ ultrafine powder in methane explosions by effectively attenuating the intensity of the explosion through significant adsorption of free radicals within the combustion zone. In contrast, fine water mist, with its efficient fire extinguishing and cooling effects achieved through instant evaporation, has emerged as a notable alternative method of extinguishment [17,18]. Yuan *et al*. [19] and Ewan *et al*. [20] studied the effects of water on coal dust explosions, revealing that water not only narrows the concentration range but also surpasses certain solid inert media in suppressing coal dust explosions.

Powder explosion suppression materials remain an important part of the explosion control field due to their convenient storage and high performance. Specifically, composite powders exhibit superior suppression efficiency compared with single powders [21]. Yan *et al*. [22] prepared composite NaHCO_3_/diatomite suppressants with a unique clustered structure by the high-pressure impact method, and the experiment proved that 60% of the suppressants effectively blocked the flame propagation of the aluminium dust explosion. In addition, Zhang *et al*. [23] synthesized boron trioxide and a composite of boron trioxide/boron trioxide zinc (ATH/ZB) powders, resulting in a remarkable enhancement of heat-absorbing capacity by adjusting the water molecule content, thereby improving the suppression performance.

Given the synergistic effects of physical and chemical explosion suppression mechanisms, studies are exploring and developing novel, highly efficient explosion suppressants that combine different performance materials [24,25]. Dry water (DW) is a unique core-shell structure composite material formed by coating water molecules with hydrophobic nanoscale silica, which stands out with its solid powder appearance, high water content, excellent fluidity and dispersibility and environmentally friendly preparation process [26]. To research the explosion inhibition performance of DW in the coal hydrogenation gasification process environment, a series of experiments were conducted in a 20 l explosion vessel by adjusting the mass percentage of DW materials and systematically analysing the effects of DW on key parameters such as explosion pressure, pressure rise rate and flame propagation velocity. In addition, we evaluated the explosion suppression performance of DW with traditional suppressants such as SiO_2_ and NH_4_H_2_PO_4_ to reveal the unique explosion suppression mechanism. These experiments not only provide evidence for utilizing DW in explosion control but also inspire new ideas for designing efficient suppressants.

## 2. Experiment

### 2.1. Experimental device and method

**Figure 1.**
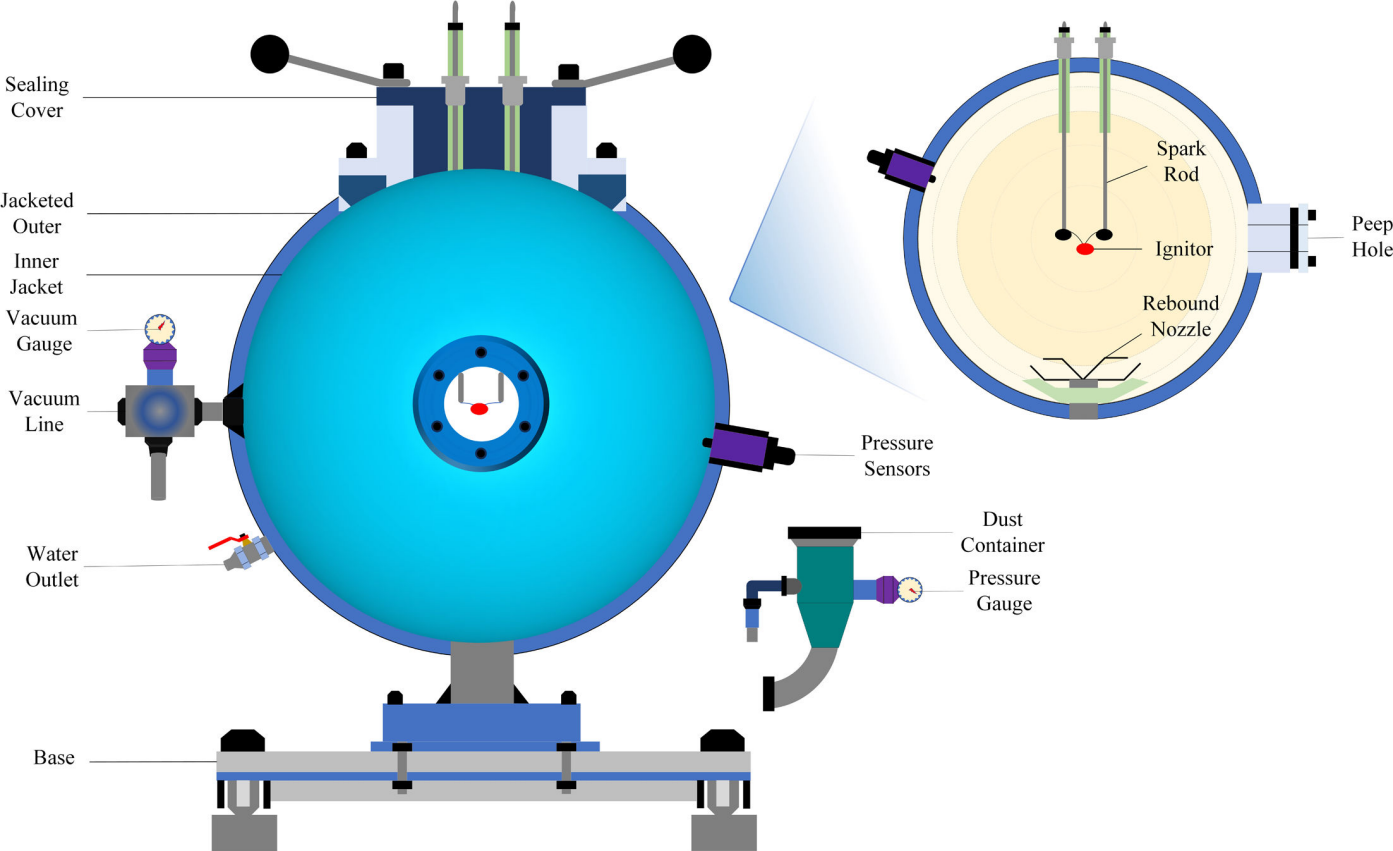
20 l spherical explosive device.

The study employed a 20 l spherical explosion device, as depicted in figure 1. The core components of the apparatus comprised an explosion sphere and a powder storage tank. The explosion sphere featured a sophisticated structure and comprehensive functionality, integrating an ignition electrode, ignitor, dispersal valve, high-precision pressure sensor, pressure gauge and an observation window. Additionally, the inlet and outlet water ports were utilized for necessary pre-treatment or cleaning operations, the sealing lid ensured the airtightness of the experimental environment, the vacuum gauge monitored the internal vacuum levels within the sphere and the gas inlet and outlet ports facilitated adjusting gas exchange and pressure regulation before and after experiments.

The experimental procedures were designed by referencing the international standards ASTM E1226 [27] and ASTM E1515 [28]. Firstly, the interior of the spherical explosion device was vacuumed to an absolute pressure of −0.06 MPa by the vacuum pump to eliminate the influence of residual gases on the experimental results. Subsequently, a high-precision gas mixing equipment was adopted to fill the ball with a preset proportion of mixed gases (2.5% H_2_ + 12.5% CO + 85% AIR) until the internal pressure reached −0.01 MPa, ensuring consistency and repeatability of the experimental environment [29]. Then, a 5 g coal dust sample was mixed with an appropriate amount of inhibitor and placed in the powder storage tank. The pressure inside the storage powder tank was adjusted to 0.2 MPa by the gas supply device to ensure that the coal dust was smoothly and efficiently sprayed into the explosive ball. After completing the above preparations, the gas mixing equipment was turned off and the spray powder switch was turned on. At this point, the high-pressure airflow carried the evenly mixed coal dust through the dispersing nozzle at high speed and sprayed it into the explosion ball, forming a dust cloud with high-turbulence characteristics. To ensure that the dust cloud fully diffused and reached a relatively stable distribution state in the explosion ball, the system set a 50 ms delay to precisely control the ignition timing. Finally, the coal dust cloud was ignited by a chemical igniter (10 J) to initiate the explosion reaction.

During the experiment, the pressure sensor was activated in real time to monitor the pressure changes during the coal dust explosion process. Simultaneously, the data signal was amplified through an integrated signal amplifier and captured by a high-precision data acquisition instrument before being transmitted to the computer for storage. The pressure sensor utilized in this experiment was the CYG508 cylindrical miniature high-pressure sensor, which featured an output voltage range of 0−5 V, an input working current of 1.5 mA, a working temperature range of −40–125°C, an accuracy of ± 0.2%, a sampling rate of 10 000 points per second and the pressure range covering 0−2 MPa. As the pressure sensor captured electrical signals rather than direct explosion pressure values, a specific conversion method was employed to more intuitively and accurately reflect the pressure dynamics during the coal dust explosion process. The conversion formula was as follows (2.1):


(2.1) $$P=0.4\cdot V_{m},$$

where *P* is the coal dust explosion pressure value, MPa; *V*_m_ is the pressure sensor voltage signal, V and 0.4 is the conversion coefficient.

### 2.2. Experimental sample

The experiment utilized coal dust with 300 mesh (*D*_50_ = 10 μm, *σ* = 2.63). To eliminate potential interference from moisture on the characteristics of coal dust explosion, all coal samples underwent a 24 h drying process in a 50°C oven prior to the experiment for complete moisture removal. DW material was utilized as an inhibitor to explore effective methods for suppressing coal dust explosions. This material possessed a unique microstructure, and its particle size was primarily distributed between 50 and 200 μm. The distinctive feature of DW lies in its shell, which consists of hydrophobic nanoscale SiO_2_ particles tightly encapsulating high-water-content water droplets (water content exceeding 90%), resulting in an overall powdery substance with a unique microscopic water storage structure. To further assess the inhibitory effect of DW, SiO_2_ and NH_4_H_2_PO_4_ were adopted as comparative reference materials, and a comparative experiment was conducted to elucidate the distinct advantages and mechanisms of DW in suppressing coal dust explosions.

## 3. Results and discussion

### 3.1. Suppression mechanism of dry water materials

**Figure 2.**
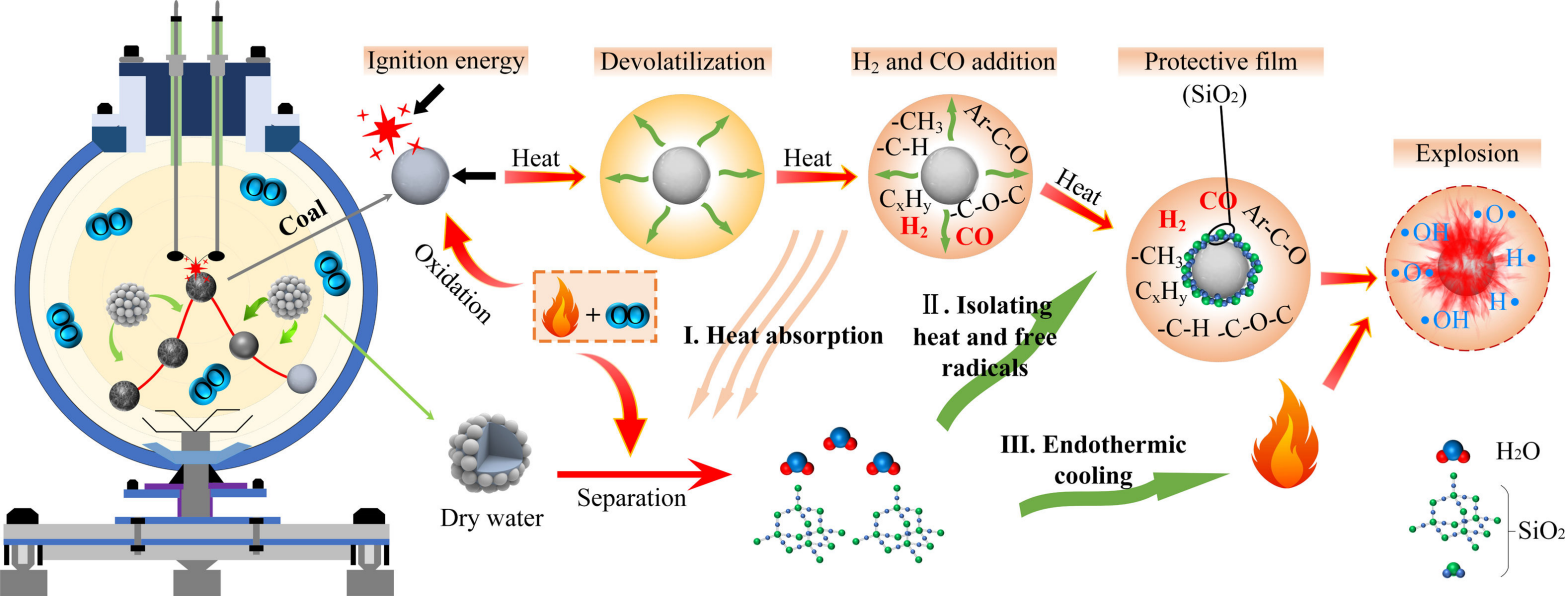
Mechanism of explosion suppression of dry water materials.

The DW exhibits an efficient result for suppressing coal dust explosions due to its unique core-shell structure and material ratio. DW appears as a white powder overall. At the microscopic scale, it is composed of approximately 90% water-phase core and 10% hydrophobic SiO_2_ nanoparticle shell. Figure 2 vividly illustrates the suppression mechanism of DW during the coal dust explosion process. After entering the explosive ball with coal dust through the nozzle to form a mixed dust cloud, a portion of the DW absorbs the ignition heat from the combustible mixed gas, undergoing separation and emitting a minor quantity of SiO_2_ and water. The moisture generated exhibits an adhesive effect on the coal dust, thereby significantly decreasing the interfacial contact between the coal dust and external gases. Consequently, this initial interaction mitigates the conditions favourable for explosion propagation. Subsequent to the ignition of the coal dust, a substantial quantity of combustible materials is liberated. The heat generated from the combustion of these materials triggers the separation of the remaining DW, yielding a considerable amount of water and SiO_2_. The heat generated by gas combustion leads to the separation of DW and the production of a substantial quantity of water and SiO_2_. The cooling effect of water weakens the intensity of the explosion reaction by dissipating heat from the environment and combustible materials through its high heat-absorbing capacity. Nanoscale SiO_2_ plays a crucial role in chain reactions leading up to an explosion: it adsorbs numerous hydroxyl radicals to reduce collision probability between free radicals and attaches itself to coal dust particle surfaces while impeding direct oxygen contact with these particles, which weakens heat transfer among them, further inhibiting the spread of an explosion [30,31]. It is particularly important that the water produced by the separation of DW evaporates as water vapour after absorbing a large amount of heat. These vapours not only reduce the oxygen concentration in the environment but also physically encase coal dust particles, effectively interrupting continuous progression towards an explosion.

### 3.2. Effect of the mass percentage of DW on explosion pressure

**Figure 3.**
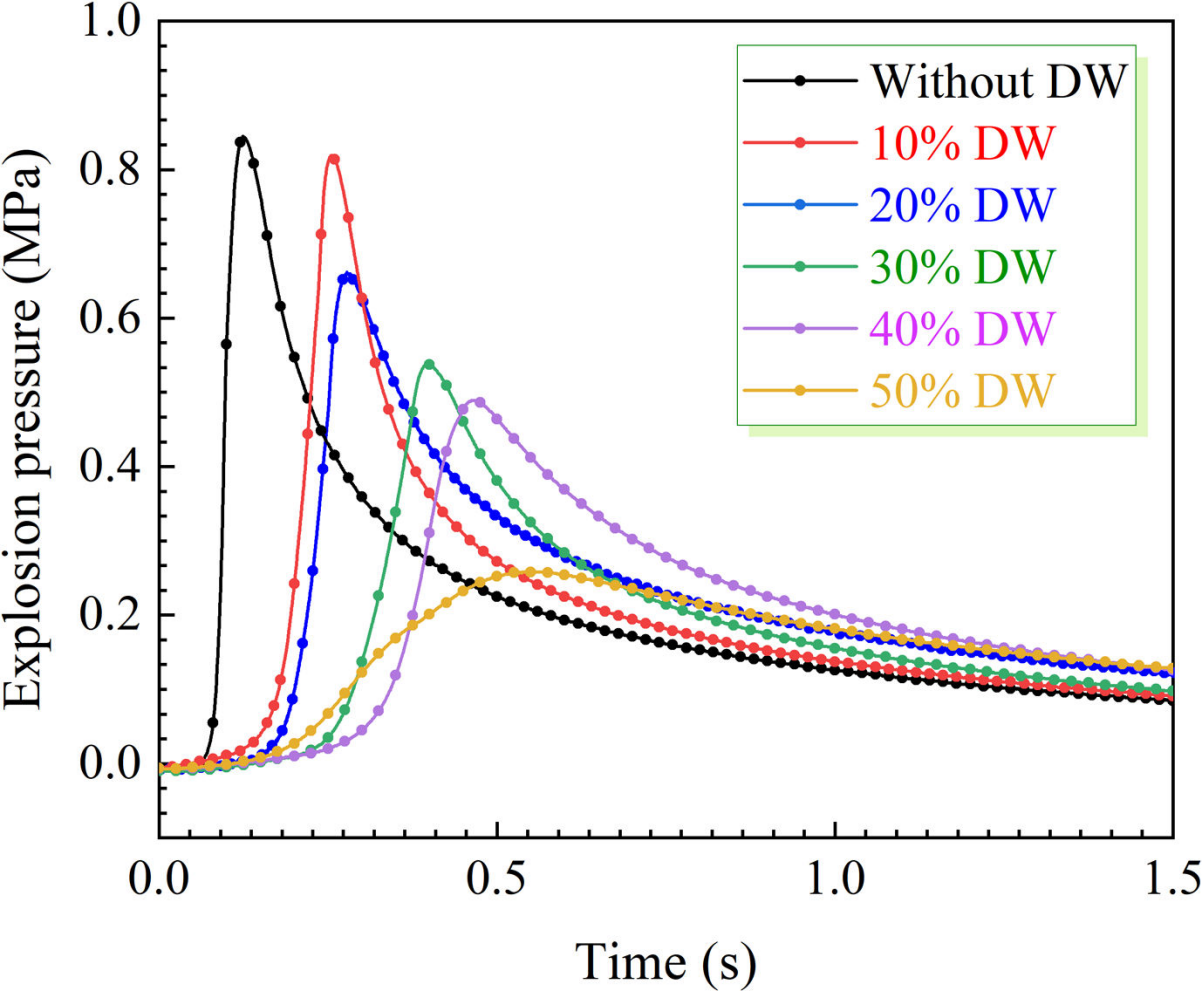
Effect of the mass percentage of DW on explosion pressure.

The graphical representation in figure 3 depicts the impact of various quality fractions of DW on the dynamic evolution of bituminous coal dust explosion pressure within a 20 l explosion device. Upon ignition of 5 g bituminous coal, the pressure rapidly escalates and reaches the maximum explosion pressure *P*_max_ = 0.844 MPa at *t* = 123.1 ms. The introduction of DW reveals two distinct trends: it significantly prolongs the time for coal dust to reach its *P*_max_ and leads to a gradual decrease in slope prior to reaching this pressure, indicating an inhibitory effect on the initial energy release rate of coal dust explosion. Specifically, the addition of 10 wt% DW results in a significant delay in the appearance time of *P*_max_, extending it to 240.7 ms. The inclusion of DW results in a significant delay of 95.5% compared with scenarios without addition, suggesting that DW effectively postpones the initial stages of the explosion process. However, the reduction in *P*_max_ by 0.025 MPa indicates that the minor amount of DW mainly affects the explosion process by prolonging the explosion time rather than directly weakening the explosion strength. Upon incremental addition of DW, the inhibitory effect on coal dust explosions intensifies, with a 50 wt% addition leading to a 69.4% reduction in peak explosion pressure to 0.258 MPa. Moreover, notable extensions in the temporal attainment of peak explosion pressures, reaching a cumulative augmentation of 431.1 ms, underscore the proficiency and potency of DW in attenuating and mitigating explosive phenomena.

**Figure 4.**
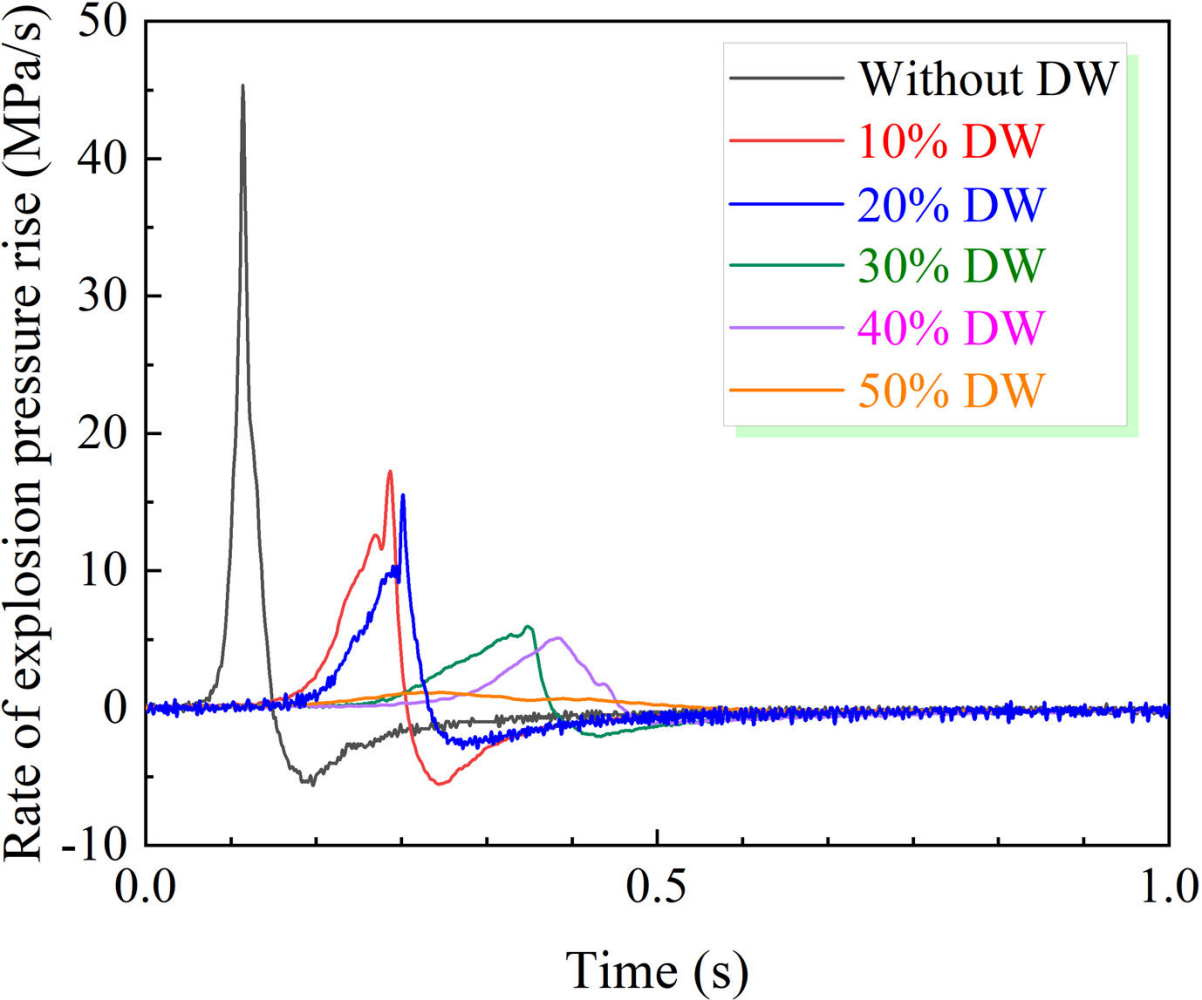
Effect of DW on the rate of explosion pressure rise.

**Figure 5.**
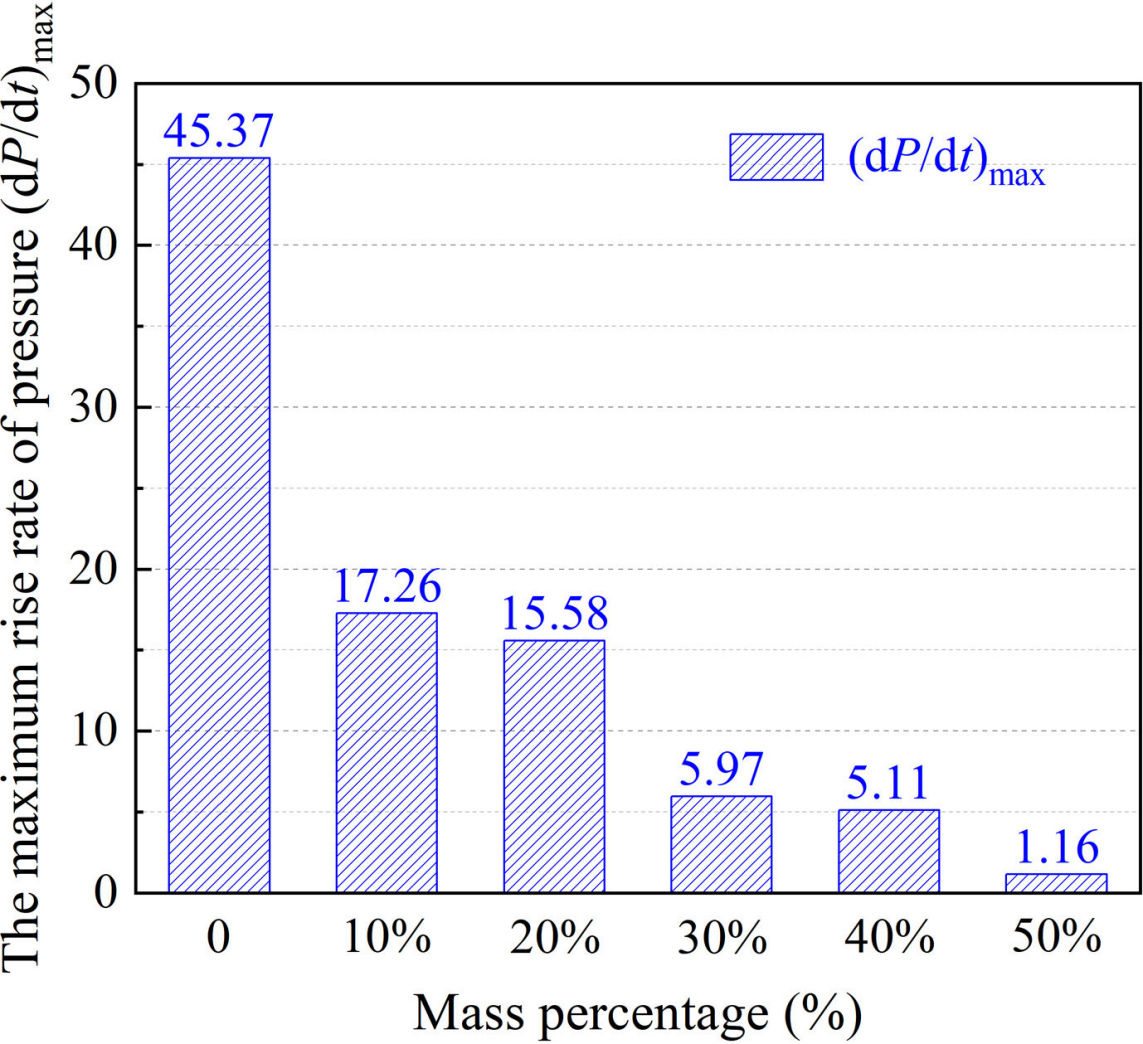
Effect of DW on the (d*P*/d*t*)_max_.

According to the experimental data in figure 4, the rise rate of coal dust explosion pressure presents a sharp and high hump without DW, which indicates that the explosion process is rapid and violent. With the increase in the amount of DW, the hump gradually decreased and widened, indicating that the intensity of the explosion was reduced and the process became gentler. As shown in figure 5, the (d*P*/d*t*)_max_ of coal dust decreases significantly with 10 wt% DW, from 45.37 to 17.26 MPa s^−1^, and the inhibition effect reaches 61.96%. The increase in the amount of DW further inhibited the rate of explosion pressure rise more obviously. Under the addition amount that reached 50 wt%, the curve of the (d*P*/d*t*)_max_ reduces to 1.16 MPa s^−1^ with a smooth, small hump shape. The significant inhibition effect of DW is attributed to its separation and generation of a substantial amount of water during the explosion. The water that breaks down during the burst absorbs a lot of energy and cools the inside of the sphere. Simultaneously, the resulting water vapour dilutes the oxygen concentration in the explosion environment, further decelerating the progression of the explosion. These two factors synergistically contribute to effectively restraining the rate of explosion pressure rises.

**Figure 6.**
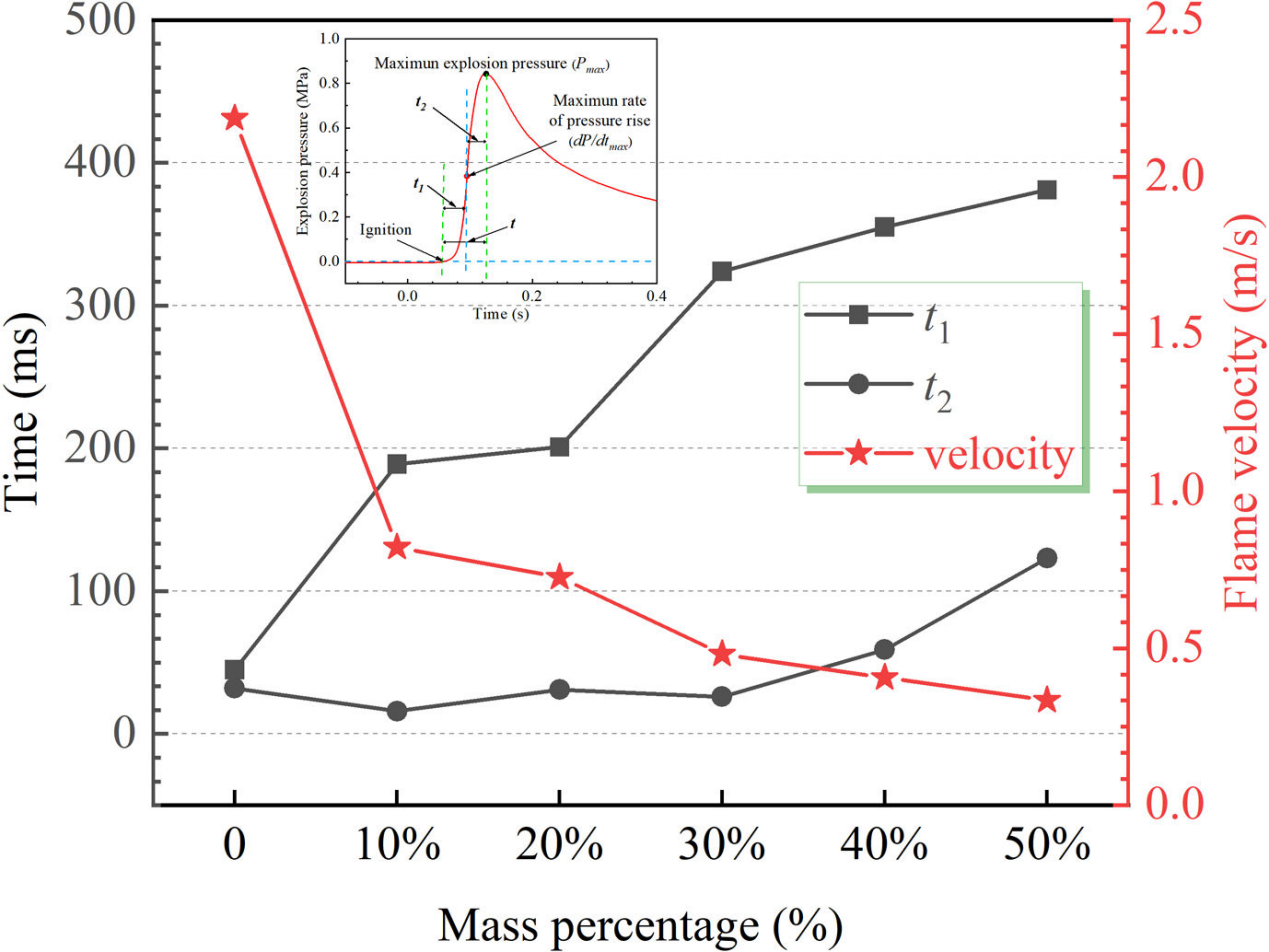
Effect of DW on explosion time and flame propagation velocity.

The diagram in figure 6 demonstrates that the ignition of the mixed dust cloud within the spherical explosion device occurs when the powder spraying delay is set to 50 ms. During this process, *t*_1_ is defined as the time interval from ignition to the (d*P*/d*t*)_max_, while *t*_2_ represents the time interval from the (d*P*/d*t*)_max_ to peak pressure appearance. The total duration of explosion (denoted as *t*) is defined as the period from ignition to peak pressure, which means *t* = *t*_1_+ *t*_2_. Furthermore, in order to characterize the energy release rate and explosion wave propagation efficiency during the explosion process, the flame propagation velocity is calculated by formula (3.1) [32]:


(3.1) $$\nu =R_{20l-sphere}/t,$$

where *v* represents the flame propagation velocity, m s^−1^; *R*_20 l-sphere_ represents the radius of the 20 l spherical explosive device, *R*_20 l-sphere_≈0.1684 m and *t* represents the time required for the explosion, s.

In figure 6, the total time required for a coal dust explosion (*t*) is 77 ms without DW, while the latencies of both *t*_1_ and *t*_2_ are relatively close, with reaching being about 40 ms. The addition of DW significantly affects the *t*_1_, with an increase in DW leading to a gradual extension of the *t*_1_. With the addition of DW reaching 30 wt%, the *t*_1_ increased significantly from 45 to 324 ms, resulting in a remarkable inhibition effect of up to 620% on the *t*_1_ phase of coal dust explosion.

Further increasing the amount of DW to 50 wt% results in an extension of *t*_1_ to 381 ms, representing an increase of 336 ms compared with the state without any addition. In contrast, the influence of DW on *t*_2_ is relatively limited. The *t*_2_ remains almost unchanged at low addition levels, with only a slight increase observed at 40 wt%. It only extended by 91 ms with the addition level reaching 50 wt%. Combining with the analysis of figure 3 reveals that the inhibitory effects exerted by DW on coal dust explosions are primarily concentrated in stage *t*_1_. The addition of DW effectively decreases the initial explosion intensity and consequently decelerates the overall explosive processes.

The introduction of DW as an inhibitor into a coal dust explosion device effectively decreases the flame propagation velocity. The explosion flame propagation velocity is reduced from 2.19 to 0.82 m s^−1^, achieving a suppression efficiency of up to 62.6% with the addition of DW reaching 10 wt%. Further research indicates that increasing the proportion of DW enhances its inhibitory effect on the explosion flame propagation velocity. Increasing the addition of DW to 50 wt% further restricts the flame propagation velocity to 0.33 m s^−1^, indicating that a higher concentration more effectively inhibits the spread of coal dust explosions. Primarily, the abundant moisture within DW is able to rapidly absorb heat during the explosion reaction process, thereby effectively reducing the temperature of combustible materials such as coal dust and reducing the possibility of reaching the critical conditions required for an explosion. Besides, the nano SiO_2_ particles on the surface of DW tightly attach to coal dust particles, forming an effective thermal barrier that blocks efficient heat transfer between combustible materials. As a result, it weakens the progress of the explosion chain reaction and effectively suppresses coal dust explosions.

### 3.3. Effects of DW on explosion characteristics with different particle sizes

**Figure 7.**
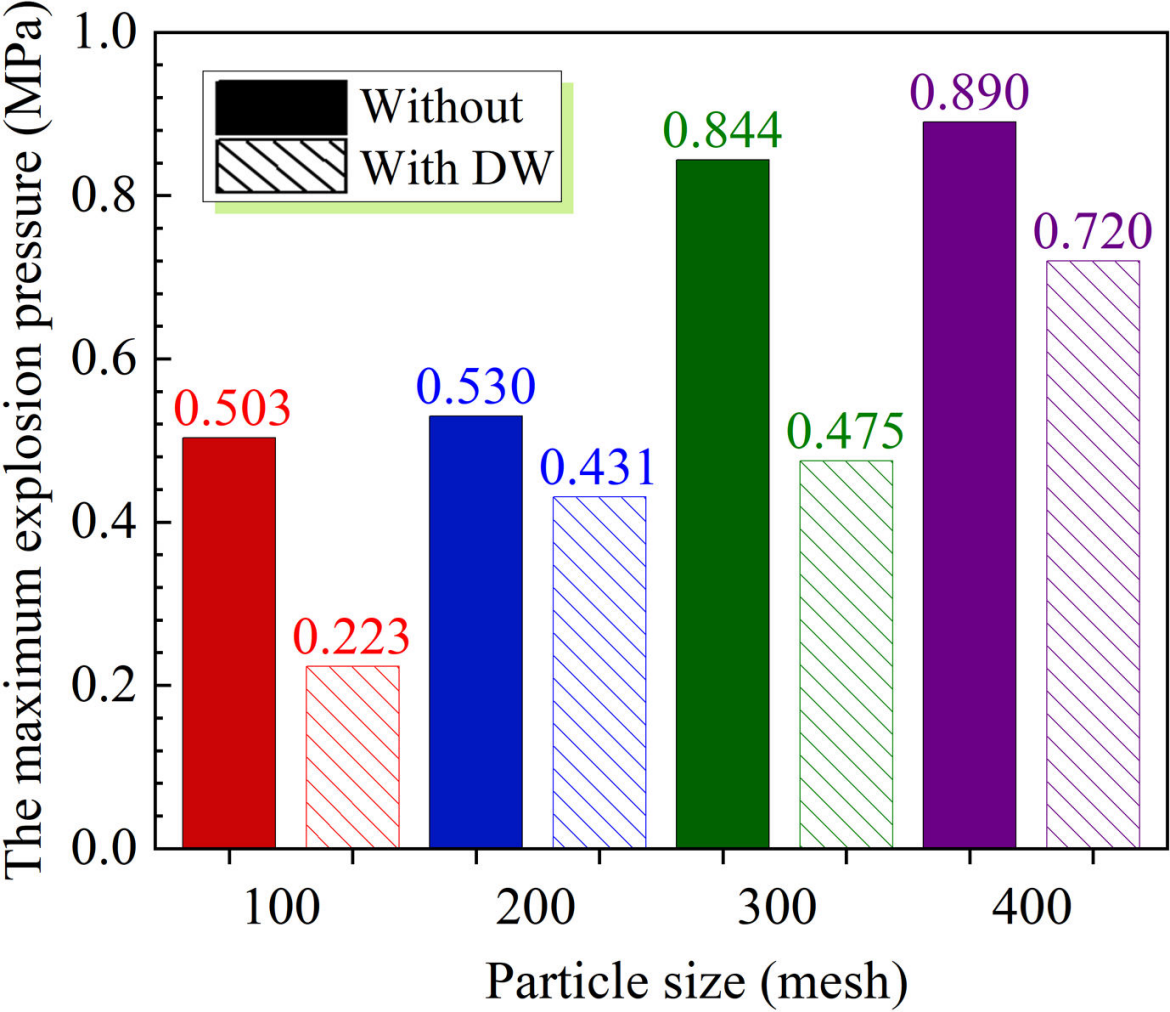
Effect of DW on *P*_max_ with different particle sizes.

As illustrated in figure 7, the maximum explosion pressure of coal dust without adding DW shows a trend of increasing gradually with the increase in particle size. Specifically, the maximum explosion pressure significantly increases to 0.89 MPa with the particle size of coal dust reaching 400 mesh. Furthermore, it is observed that the maximum explosion pressure of coal dust of all particle sizes decreases greatly with the addition of 30 wt% DW to coal dust. The DW material reduces the maximum explosion pressure from 0.503 to 0.223 MPa with coal dust particle size of 100 mesh, representing a suppression efficiency of 55.6%. Additionally, the DW material demonstrates a notable inhibitory effect on the explosion pressure of 300 mesh coal dust with a decrease of 43.7%. However, for 200 mesh and 400 mesh coal dust, although the maximum explosion pressure decreases with the addition of DW, the reductions are relatively modest, at 0.099 and 0.17 MPa, respectively.

**Figure 8.**
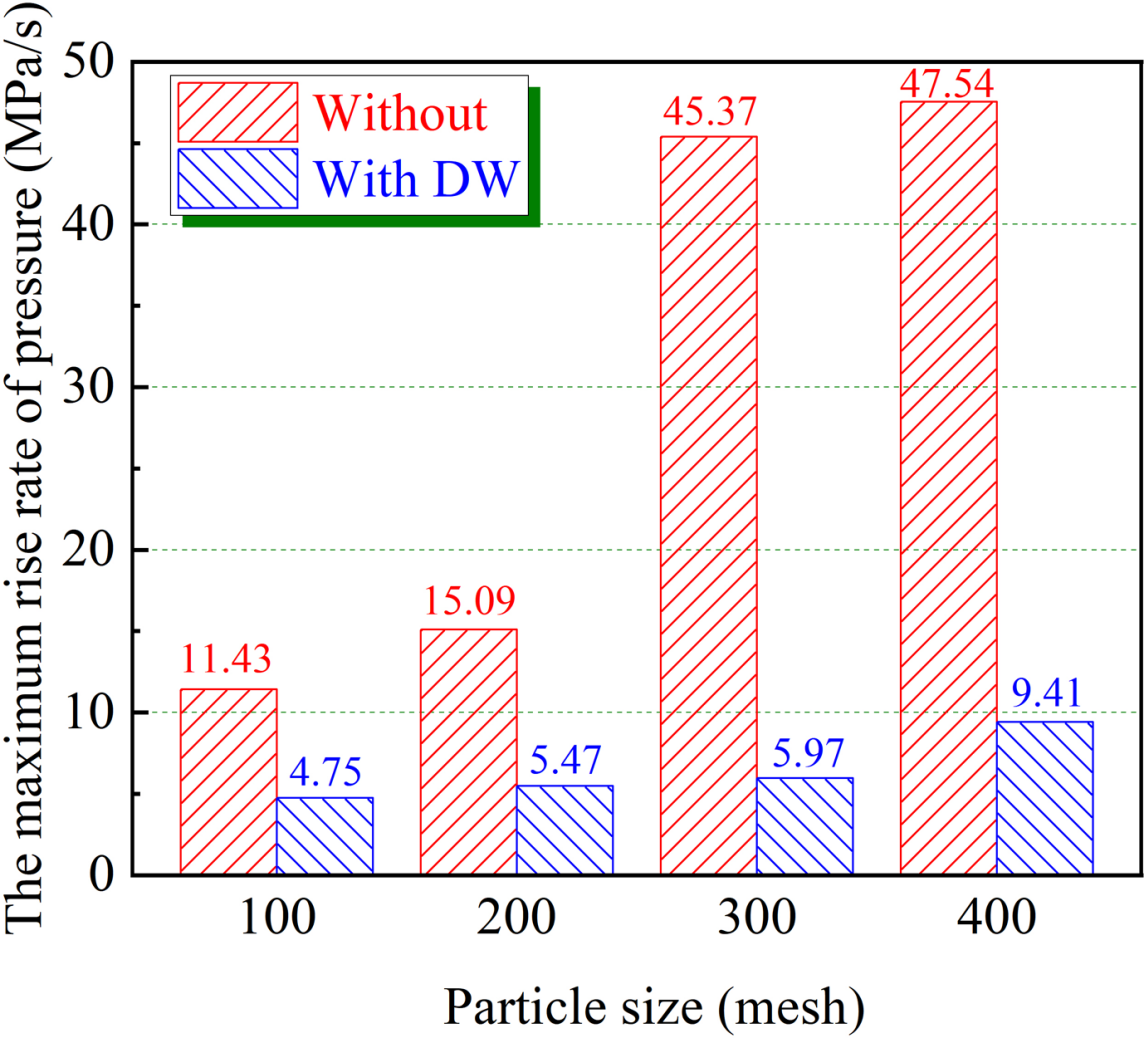
Effect of DW on (d*P*/d*t*)_max_ with different particle sizes.

**Figure 9.**
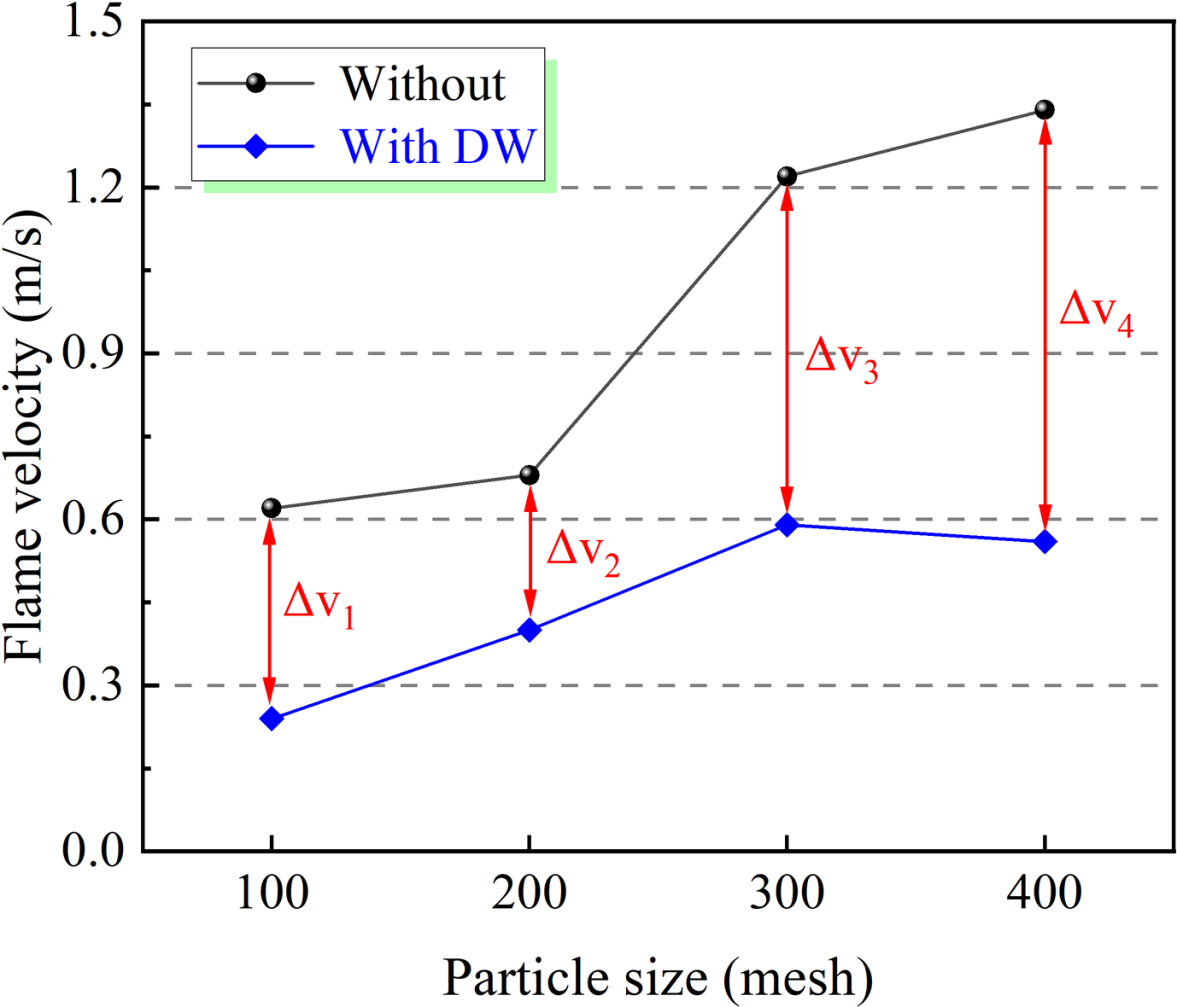
Effect of DW on flame propagation velocity.

As shown in figure 8, with the decrease in coal dust particle size, the maximum rise rate of coal dust explosion pressure increases from 11.43 MPa s^-1^ at 100 mesh to 47.54 MPa s^-1^ at 400 mesh. It is worth noting that the addition of DW has an excellent inhibition effect on the (d*P*/d*t*)_max_ of coal dust. Regardless of the particle size of coal dust, the (d*P*/d*t*)_max_ of coal dust is effectively controlled below 10 MPa s^-1^. Among them, the DW has a particularly prominent inhibition effect on 300 mesh coal dust, which makes the (d*P*/d*t*)_max_ of coal dust decrease from 45.37 to 5.97 MPa s^-1^, and the inhibition rate is as high as 86.8%. Further, as shown in figure 9, the variation trend of the flame propagation velocity of coal dust explosion is similar to that of *P*_max_ and (d*P*/d*t*)_max_, and both gradually increased with the decrease in particle size. In particular, 400 mesh coal dust has an explosion flame propagation velocity of 1.34 m s^−1^. However, the flame propagation velocity of coal dust with different particle sizes is effectively slowed down with the addition of DW. Specifically, the explosion flame propagation velocity of 400 mesh coal dust is reduced by 0.78 m s^−1^, while that of 300 mesh coal dust is significantly reduced from 1.22 to 0.55 m s^−1^, with an inhibition effect of 54.9%.

### 3.4. Effects of inhibitor types on explosion characteristics

**Figure 10.**
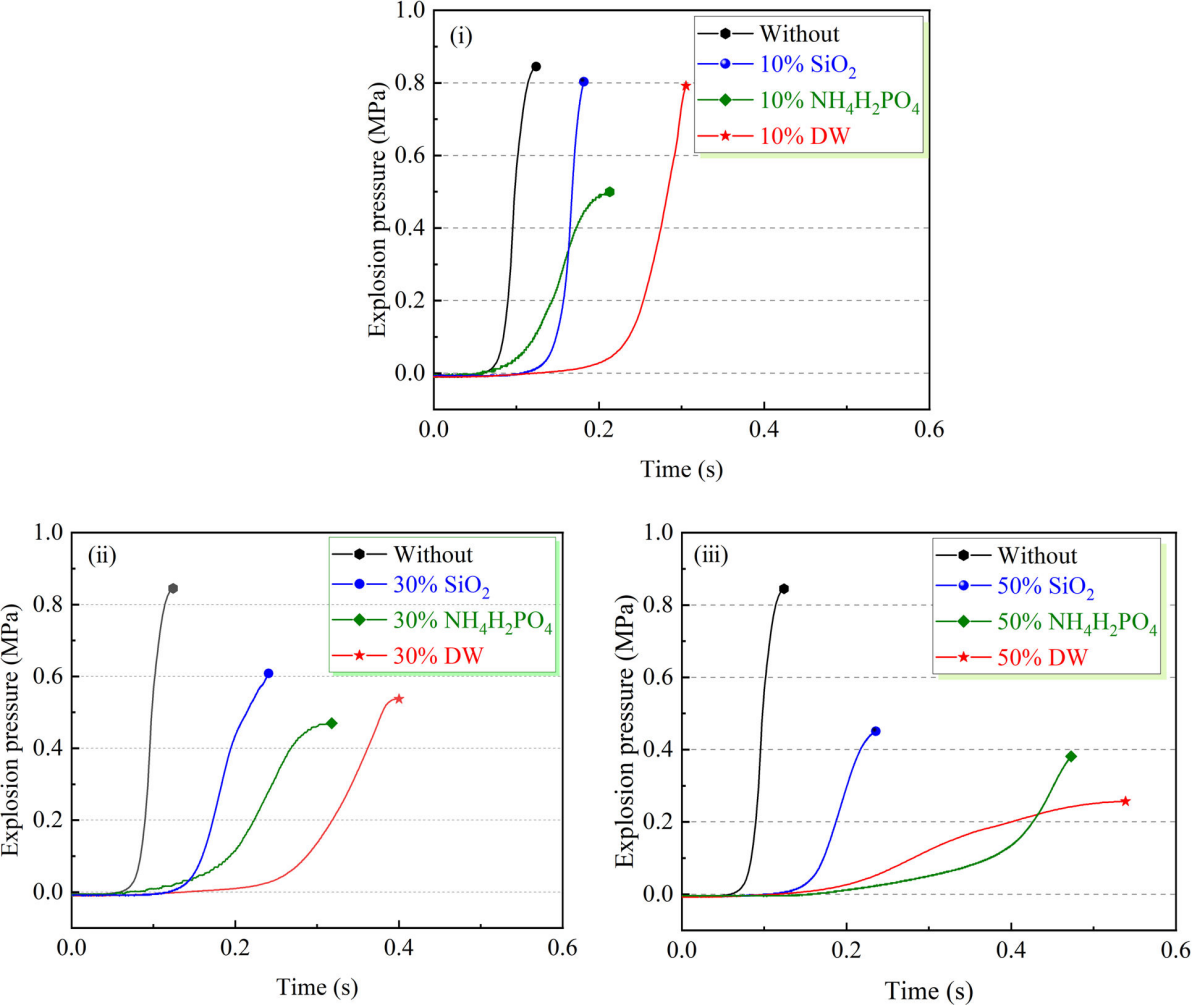
(i)−(iii) Effect of inhibitor types on explosion pressure.

To study the inhibitory effects of diverse inhibitors on coal dust explosions, SiO_2_, NH_4_H_2_PO_4_ and DW are adopted into coal dust samples in specific, predetermined proportions as inhibitors. The dynamic evolution of the pressure curve during the explosion events is meticulously monitored. The experimental results obtained unequivocally demonstrate the substantial impact of each inhibitor on the characteristics of coal dust explosion pressure. Notably, as shown in figure 10(i), when the inhibitors reach 10 wt% of the total coal dust mass, NH_4_H_2_PO_4_ exhibits remarkable suppression capabilities, decreasing the peak explosion pressure from 0.844 to 0.495 MPa, yielding a substantial inhibition rate of 41.3%. In contrast, while DW and SiO_2_ displayed limited reductions in the peak explosion pressure, DW is effective in prolonging the time required for the coal dust to attain peak pressure, thereby delaying the explosion process to a certain degree. As shown in figure 10(ii), upon increasing the inhibitor concentration to 30 wt%, NH_4_H_2_PO_4_ maintains its superior explosion suppression performance, further decrementing the initial peak pressure to 0.461 MPa, with an efficacious inhibition rate of 45.4%. At this concentration, SiO_2_ exhibits a relatively weaker inhibitory effect on the peak pressure, reducing it to only 0.217 MPa. DW demonstrates moderate inhibitory action, lowering *P*_max_ to 0.537 MPa with an inhibition rate of 36.4%. According to figure 10(iii), as the inhibitor concentration is further increased to 50 wt%, DW surpasses NH_4_H_2_PO_4_ in inhibitory effectiveness, becoming the most potent inhibitor. DW significantly decreased the *P*_max_ to 0.258 MPa, which represents a substantial reduction of 0.586 MPa compared with the uninhibited condition of 0.844 MPa, yielding an inhibition rate of 69.4%. Conversely, NH_4_H_2_PO_4_ and SiO_2_ reduce the explosion peak pressure by 0.451 and 0.384 MPa, respectively. It is particularly noteworthy that DW not only excels in suppressing the maximum explosion pressure but also possesses a unique advantage in extending the explosion, which is crucial for slowing down the explosion reaction rate and effectively mitigating the associated damage.

**Figure 11.**
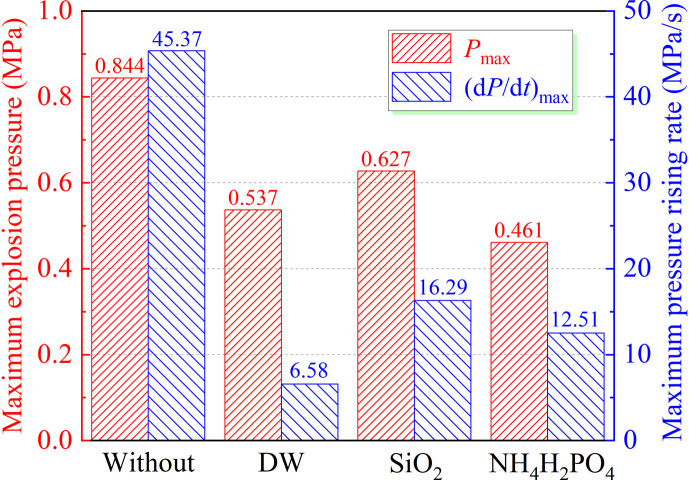
Effect of inhibitor types on *P*_max_ and (d*P*/d*t*)_max_.

Figure 11 shows the effect of different inhibitors on the (d*P*/d*t*)_max_ of coal dust. The experimental results demonstrate a significant decrease in the rise rate with the addition of SiO_2_, NH_4_H_2_PO_4_ and DW. Among them, NH_4_H_2_PO_4_ exhibits the most effective inhibition on the *P*_max_, reducing the (d*P*/d*t*)_max_ from 45.39 to 12.51 MPa s^-1^, achieving a suppression effect of 72.4%. In contrast, DW performs better in decreasing the pressure rise rate to 6.58 MPa s^-1^, resulting in an impressive suppression effect of 85.5%. It is worth noting that SiO_2_ has limited efficacy in inhibiting coal dust explosions and fails to effectively retard the rapid development of explosion processes. The performance of DW in inhibiting coal dust explosion characteristics is significantly superior to that of NH_4_H_2_PO_4_ and SiO_2_, considering the key parameters of explosion pressure and rise rate.

**Figure 12.**
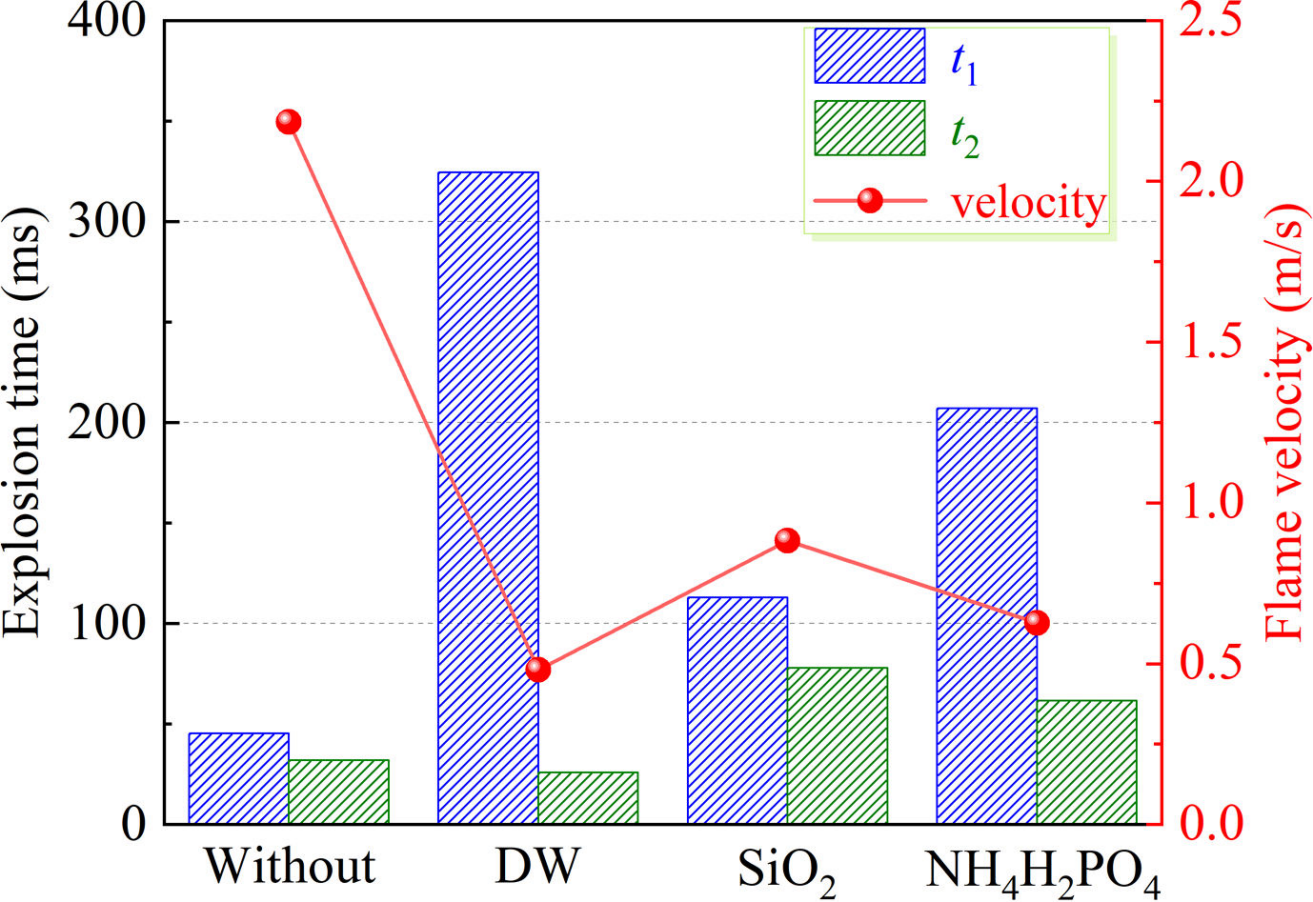
Effect of inhibitor types on explosion time and flame propagation velocity.

Figure 12 investigates the impact of various inhibitors on the duration of explosion and flame propagation velocity in bituminous coal dust. The study reveals significant variations among different inhibitors. In the absence of any inhibitors, the coal dust explosion process primarily comprises stages *t*_1_ and *t*_2_, lasting for 45 and 32 ms, respectively.

Both SiO_2_ and NH_4_H_2_PO_4_ moderately prolong these two stages. Specifically, the addition of SiO_2_ extends *t*_1_ to 112.9 ms and *t*_2_ to 77.8 ms. However, its efficacy is comparatively limited compared with other inhibitors. Conversely, DW exhibits more pronounced effects in attenuating overall explosive power by extending *t*_1_ to 324.5 ms and achieving a time delay rate of 621.1%. The combined influence of DW on the maximum explosion pressure rise rate indicates a primary focus on stage *t*_1_. Furthermore, under the addition of 30 wt%, DW reduces flame propagation velocity from 2.19 to 0.48 m s^-1^, resulting in an effective suppression effect of 78.1%. In contrast, NH_4_H_2_PO_4_ reduces flame propagation velocity to 0.63 m s^-1^ but exhibits weaker suppression effects than DW. The flame propagation velocity reduction of SiO_2_ mainly relies on physical inhibition, which exhibits a more limited capability with only a decrease of 1.31 m s^−1^.

## 4. Conclusions

The DW material has proven effective in suppressing coal dust explosions. Adding 10 wt% DW doubles the time it takes for the *P*_max_ to reach 240.7 ms and reduces the (d*P*/d*t*)_max_ increase by 61.96%. Increasing DW to 50 wt% significantly reduces the *P*_max_ by 69.4% and almost completely suppresses the (d*P*/d*t*)_max_ increase, which is reduced to 1.16 MPa s^−1^. Additionally, adding 50 wt% of DW significantly slows down the spread of the explosion, reducing the flame propagation velocity to 0.33 m s^−1^.

Adding 30 wt% DW results in a notable reduction in the peak pressure of 300 mesh coal dust by 0.369 MPa. The DW exhibited substantial inhibitory capabilities, effectively limiting the (d*P*/d*t*)_max_ for four distinct particle sizes of coal dust to below 10 MPa s^−1^. Furthermore, the DW possesses the ability to significantly diminish the propagation velocity of explosion flame, which reduces the flame propagation velocity of 300 mesh coal dust by 0.67 m s^−1^ and that of 400 mesh coal dust by 0.78 m s^−1^.

NH_4_H_2_PO_4_ demonstrates significant suppression capabilities at lower concentrations. Adding 30 wt% of NH_4_H_2_PO_4_ reduced the *P*_max_ of coal dust explosion by approximately 45.4%, lowering it from 0.844 to 0.461 MPa. The addition of DW material decreased the *P*_max_ of coal dust to 0.537 MPa, achieving a suppression rate of 36.4%. It significantly reduced the (d*P*/d*t*)_max_ from 45.39 to 6.58 MPa s^-1^, resulting in an impressive suppression rate of 85.5%. In contrast, SiO_2_ exhibited weaker inhibitory effects, only reducing the *P*_max_ by 0.217 MPa. DW exhibits moderate to strong inhibition with increasing concentration. Additionally, it notably reduced the flame propagation velocity from 2.19 to 0.48 m s^−1^ with the addition of 30 wt%, demonstrating a suppression efficiency of 78.1%.

## Data Availability

Data is available online [33].
